# The Impaired Function of Macrophages Induced by Strenuous Exercise Could Not Be Ameliorated by BCAA Supplementation

**DOI:** 10.3390/nu7105425

**Published:** 2015-10-21

**Authors:** Weihua Xiao, Peijie Chen, Xiaoguang Liu, Linlin Zhao

**Affiliations:** Department of Sports Science, Shanghai University of Sport, Shanghai 200438, China; liuxiaoguang_sport@163.com (X.L.); linlinzhao666@163.com (L.Z.)

**Keywords:** strenuous exercise, branched-chain amino acid, macrophages, function

## Abstract

The aim of this study was to evaluate the effect of strenuous exercise on the functions of peritoneal macrophages in rats and to test the hypothesis that branched-chain amino acid (BCAA) supplementation will be beneficial to the macrophages of rats from strenuous exercise. Forty male Wistar rats were randomly divided into five groups: (C) Control, E) Exercise, (E1) Exercise with one week to recover, (ES) Exercise + Supplementation and (ES1) Exercise + Supplementation with 1 week to recover. All rats except those of the sedentary control were subjected to four weeks of strenuous exercise. Blood hemoglobin, serum testosterone and BCAA levels were tested. Peritoneal macrophages functions were also determined at the same time. The data showed that hemoglobin, testosterone, BCAA levels, and body weight in group E decreased significantly as compared with that of group C. Meanwhile, phagocytosis capacity (decreased by 17.07%, *p* = 0.031), reactive oxygen species (ROS) production (decreased by 26%, *p* = 0.003) and MHC II mRNA (decreased by 22%, *p* = 0.041) of macrophages decreased in the strenuous exercise group as compared with group C. However, the chemotaxis of macrophages did not change significantly. In addition, BCAA supplementation could slightly increase the serum BCAA levels of rats from strenuous exercise (increased by 6.70%, *p* > 0.05). Moreover, the body weight, the blood hemoglobin, the serum testosterone and the function of peritoneal macrophages in group ES did not change significantly as compared with group E. These results suggest that long-term intensive exercise impairs the function of macrophages, which is essential for microbicidal capability. This may represent a novel mechanism of immunosuppression induced by strenuous exercise. Moreover, the impaired function of macrophage induced by strenuous exercise could not be ameliorated by BCAA supplementation in the dosing and timing used for this study.

## 1. Introduction

Exercise enhances or reduces immune functions depending on its frequency, duration and intensity. Regular physical activity is known to enhance immune functions leading to a decrease in the occurrence of infections. On the other hand, heavy or exhaustive exercise increases the susceptibility to infections [1,2,3]. Exercise increases or decreases the occurrence of infections that may be related to the changes of macrophage functions [4,5]. Monocytes/macrophages are considered to be the frontline of immunological defense against pathogens. These cells have prominent roles such as Ag presentation, chemotaxis, phagocytic, microbicidal, tumoricidal and secretory functions, as well as, innate immunity, by initiating inflammatory and immune responses [6]. Although some studies focus on the relationship between exercise and macrophages, little attention has been paid to the effects of long-term intensive exercise on macrophage functions.

Branched-chain amino acid (BCAA) participates in skeletal muscle protein synthesis and can be used as energy substrates during physical exercise [7,8]. Therefore, since the 1980s, there has been high interest in BCAA by sports nutrition scientists. And now BCAA has been commonly used by Chinese athletes as nutritional supplements. What benefits can we get from the BCAA supplementation? Studies have shown that BCAA supplementation before and after exercise has beneficial effects for decreasing exercise-induced muscle damage and promoting muscle-protein synthesis [9,10]. However, many researchers have not been able to confirm that BCAA supplementation can enhance sports performance [11]. BCAAs act as donors of nitrogen and carbon skeleton for the synthesis of other amino acids, e.g., glutamine that are important in supporting immune cell function [12,13]. Thus, in recent years investigators have changed their research target and focused on the effects of BCAA on immune system. Cell culture and animal feeding studies indicate that an adequate supply of BCAA is necessary to support efficient immune function [14,15]. Conversely, insufficient availability of BCAA impairs some aspects of immune function, including killer-cell activity and lymphocyte proliferation [14,15]. However, many aspects of BCAAs and their effect on immune function have received little or no attention. For instance, we still do not know the effect of BCAA supplementation on the macrophage functions of rats from strenuous exercise.

Consequently, the aim of this study is to evaluate the effect of strenuous exercise on the functions of peritoneal macrophages in rats, and to test the hypothesis that BCAA supplementation will be beneficial to the macrophages of rats from strenuous exercise.

## 2. Materials and Methods

### 2.1. Animals

**Figure 1:**
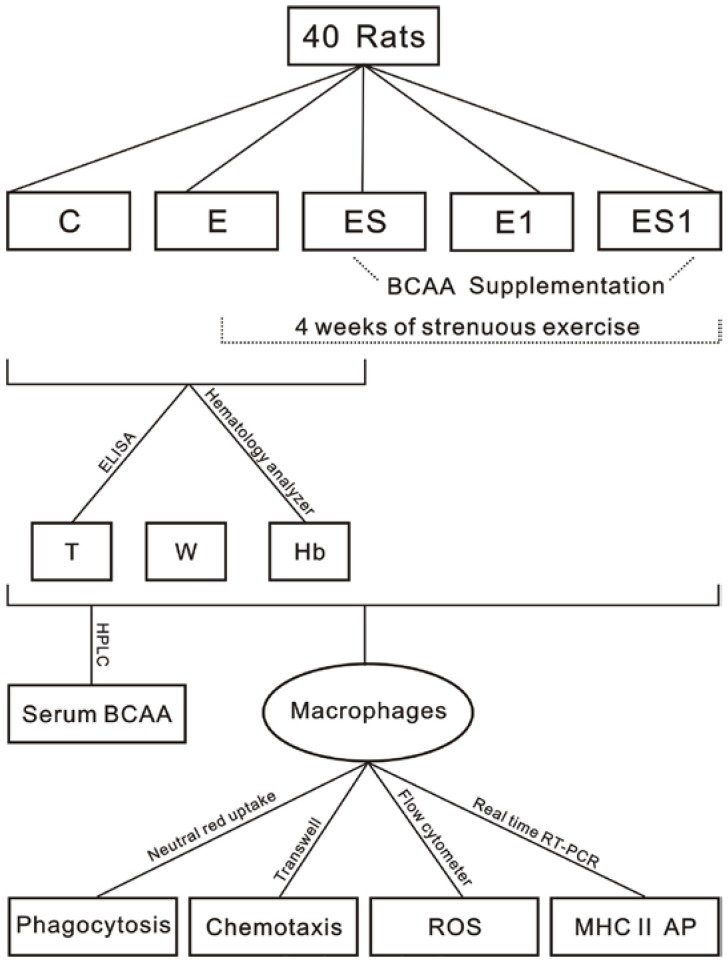
Schematic representation of the experimental program. Group C, Control; E, Exercise; E1, Exercise with 1 week to recover; ES, Exercise + Supplementation; ES1, Exercise + Supplementation with 1 week to recover. T, testosterone; W, weight; Hb, hemoglobin; BCAA, branched-chain amino acid; ROS, Reactive oxygen species; AP, antigen presentation.

Forty male Wistar rats (mean weight 207 ± 10.6 g) were purchased from Shanghai SLAC laboratory animal center and were fed for one week acclimatization phase (environmental temperature 20–25 °C with a 12 h light/dark cycle and free access to standard pellets and drinking water). The rats were randomly divided into 5 groups (*n* = 8): (C) Control, (E) Exercise, (E1) Exercise with 1 week to recover, (ES) Exercise + Supplementation and (ES1) Exercise + Supplementation with 1 week to recover. All groups except group C were subjected to 4 weeks of strenuous exercise. In order to avoid the acute effect of exercise, the rats of group E and ES were killed at 36 h after the last training. The schematic representation of the experimental program is shown in Figure 1.

### 2.2. Exercise Program

The exercise program was based on a previously validated protocol [16]. All rats except those in group C underwent daily running training sessions on a treadmill. The treadmill had different lanes to serve as corridors for each animal. In order to avoid the stimulation of the immune system, electric shock was not used in this study. We used our hands to ensure the animals ran effectively. The protocol included a 2-week progressive training program, starting with a 10-min running session at 6 m/min and increasing gradually to steady-state 60-min running at 36 m/min. Thereafter, animals were trained at this level 5 days a week for 4 weeks. At the same time, the sedentary control group was handled and exposed to the treadmill to control for stress of treadmill environment. The experimental protocol was approved by the Ethics Review Committee for Animal Experimentation of Shanghai University of Sports (Code number: 2014025).

### 2.3. BCAA Supplementation

The diet of rats was elaborated according to the recommendations of the American Institute of Nutrition (AIN-93M) for the maintenance of adult rodents (Research Diets, Inc., New Brunswick, USA) [17]. The composition of the experimental diets is shown in Table 1. Rats were fed with BCAA mixture (600 mg/kg body weight/day, consist of 46% leucine, 28% valine, and 23% isoleucine, Ajinomoto, Tokyo, Japan) (group ES and ES1) or saline (group C, E and E1) by gavage administration post-exercise and maintained for 4 weeks. The dose and delivery method of BCAA was determined based on previous studies [18,19].

**Table 1:** Composition of the experimental diets * (g/kg).

Ingredients	g	kcal
Casein	140.000	560
l-Cystine	1.800	7.2
Corn Starch	495.692	1983
Maltodextrin	125	500
Sucrose	100.000	400
Cellulose	50.000	0
Soybean Oil	40.000	360
*t*-Butylhydroquinone	0.008	0
Mineral mix	35.000	0
Vitamin mix	10.000	40
Choline bitartrate	2.500	0
Total	1000	3850

* The diet was elaborated according to the recommendations of the American; Institute of Nutrition (AIN-93M) for the maintenance of adult rodents [17].

### 2.4. Biochemical Analyses

Blood sample (3 mL) was taken from the fossa orbitalis venous plexus of rats. Blood hemoglobin, serum testosterone and serum BCAA level were determined. Blood hemoglobin was measured by hematology analyzer (Sysmex, Japan). Serum testosterone level was assessed using a commercial ELISA kit (AssayPro, St. Charles, MO, USA) according to the manufacturer’s instructions. All samples and standards were measured in duplicate. BCAA levels in the serum were measured using high-performance liquid chromatography (HPLC, Hitachi, Ltd., Tokyo, Japan) according to the method by Deyl *et al.* [20].

### 2.5. Peritoneal Macrophages Preparation

As reported in our previous study [5], the MФs (peritoneal macrophages) were removed by peritoneal lavage using RPMI 1640 (GIBCO, Carlsbad, CA, USA). The cells were washed by centrifugation, resuspended in RPMI 1640 with 10% fetal bovine serum (GIBCO, Carlsbad, CA, USA), plus 1% penicillin-streptomycin solution, and then placed in 6-well tissue culture microplates. Plates were incubated for 2 h at 37 °C in a humidified atmosphere of 5% CO_2_. After the removal of non-adherent cells, the adherent cells were detached by treatment with 0.25% Trypsin and suspended in RPMI 1640 at a concentration of 2 × 10^6^ cells/mL. Cell viability was checked with the Trypan blue dye and was >96%. Cell purity checked by the Giemsa dye test was >98%.

### 2.6. Chemotaxis Assay

Following Yang *et al.* [21] and Novak *et al.* [22], with little modification, the macrophages were washed twice in serum-free RPMI 1640 and resuspended at 1 × 10^6^ cells/mL. 100 μL cells were added into the upper chambers of a 24-well transwell plate with 8-μm pore size polycarbonate filters (Costar, Corning, NY, USA). The plate was equilibrated at 37 °C in a 5% CO_2_ cell culture incubator for 30 min. 600 μL of the serum-free RPMI 1640 (MCP-1, 10 ng/mL, Sigma-Aldrich, St. Louis, MO, USA) was added into the lower chambers of the transwells to induce migration. After 2 h at 37 °C in a 5% CO_2_ cell culture incubator, the cells remaining in the upper chambers were wiped off with a cotton swab. Migrated cells attached to the lower surface of the filters were fixed with 75% ethanol for 30 min, washed with water, and stained with hematoxylin. The number of migrated cells was counted under microscope. For each sample, cells in 5 randomly picked fields under 200× magnification were counted. Data were expressed relative to control group cell migration.

### 2.7. Phagocytosis Assay

The uptake of the neutral red by macrophages was measured following Long *et al.* [23] with the following modifications. The cell suspension (2 × 10^6^ cells/mL) was incubated in a 96-well flat-bottomed microtiter plate 100 µL/well for 2 h at 37 °C in a 5% CO_2_ cell incubator. After one wash with warm PBS (pH 7.2 to 7.4), 200 µL of 0.1% neutral red (Amersco, Solon, OH, USA) solution in PBS was added. To minimize crystal formation during the neutral red assay, the dye solution was incubated overnight at 37 °C and sterile filtered before use. After 30 min of incubation of the culture plates at 37 °C, neutral red solution was aspirated, and each well was thrice carefully rinsed with PBS. Finally, the intracellular dye was extracted with 200 µL of a mixture of 100% ethanol and 99.9% acetic acid (1:1 *v*/*v*). The mixtures were mixed fully and evaluated at a wavelength of 550 nm on a Bio-Rad 550 microplate reader (Bio-Rad Laboratories, Hercules, CA, USA). The absorbance represented phagocytosis by macrophages.

### 2.8. Reactive Oxygen Species Determination

Following Bae *et al.* [24], with little modification (no stimulus was added to the cell suspension to induce macrophage activation), the macrophages (5 × 10^5^ cells) were incubated with 2′,7′-dichlorofluorescein diacetate (DCFH-DA; Molecular Probes) for 20 min. The fluorescence intensity was analyzed by flow cytometry using a Coulter EPICS XL^TM^ flow cytometer with the System II^TM^ software (Beckman Coulter, Fullerton, CA, USA). The level of ROS was expressed as relative fluorescence intensities generated by counting 10,000 cells.

### 2.9. Real Time reverse-transcription polymerase chain reaction (Real Time RT-PCR)

Total RNA of macrophages was isolated using a modified guanidinium isothiocyanate-CsCl method [25]. RNA was reverse transcribed into cDNA using the Revertaid^TM^ First Strand cDNA Synthesis Kit from Fermentas. Quantitative PCR was carried out in triplicates in reactions consisting of 12.5 µL 2×Maxima SYBR Green/ROX qPCR Master mix (Thermo Scientific), 1 µL cDNA, nuclease-free water and 300 nM of each primer [26]. Using Primer Express software (Applied Biosystems), we designed the following primers for the present study: β-actin (forword: 5’-GGA GAT TAC TGC CCT GGC TCC TA-3’; reverse: 5’-GAC TCA TCG TAC TCC TGC TTG CTG-3’) and MHC II α chain (forword: 5’-AGA GAC CAT CTG GAG ACT TG-3’; reverse: 5’-CAT CTG GGG TGT TGT TGG A-3’). Amplifications were performed on a StepOne Plus™ PCR-Cycler (Life Technologies) with the following parameters: activation at 95 °C for 10 min, 40 cycles of denaturation at 95 °C for 15 s, and annealing/extension at 60 °C for 1 min. The threshold cycle (CT, the number of cycles to reach threshold of detection) was determined for each reaction, and the levels of the target mRNAs were quantified relatively to the level of the housekeeping gene β-actin using 2^−ΔΔCT^ method [27].

### 2.10. Statistical Analysis

All values are expressed as mean ± SD( Standard Deviation), and statistical significance was set at *p* < 0.05. Mean values were compared between groups by ANOVA(Analysis of Variance) with the LSD(Least Significant Difference) method as a *post hoc* test. Data were analyzed using SPSS 19.0 for windows.

## 3. Results

### 3.1. Body Weight, Hemoglobin and Testosterone Levels

The mean final body weight and the mean concentrations of blood hemoglobin and serum testosterone are presented in Table 2. The weight of the rats of the strenuous exercise group was significantly lower than that of the sedentary control group (decreased by 13.86%, *p* = 0.000). In addition, blood assay showed that blood hemoglobin and serum testosterone in strenuous exercise group decreased significantly as compared with the sedentary control group (decreased by 9.27%, *p* = 0.005; 31.40%, *p* = 0.001; respectively). Furthermore, body weight, blood hemoglobin and serum testosterone in group ES were still significantly lower than that of group C. There was no significant difference between group ES and E (*p* > 0.05).

**Table 2:** Body Weight, Hemoglobin and Testosterone levels.

Group	Body Weight (g)	Hemoglobin (g/L)	Testosterone (ng/mL)
C	318.25 ± 11.57	142.88 ± 4.16	4.49 ± 0.55
E	274.13 ± 17.68 ^**^	129.63 ± 10.56 ^**^	3.08 ± 0.80 ^**^
ES	277.75 ± 14.48 ^**^	132.87 ± 9.50 ^*^	2.81 ± 0.73 ^**^

Values are means ± Standard Deviation (SD); * *p* < 0.05, ** *p* < 0.01 from control group. C, Control; E, Exercise; ES, Exercise + Supplementation.

### 3.2. Serum BCAA Levels

**Figure 2:**
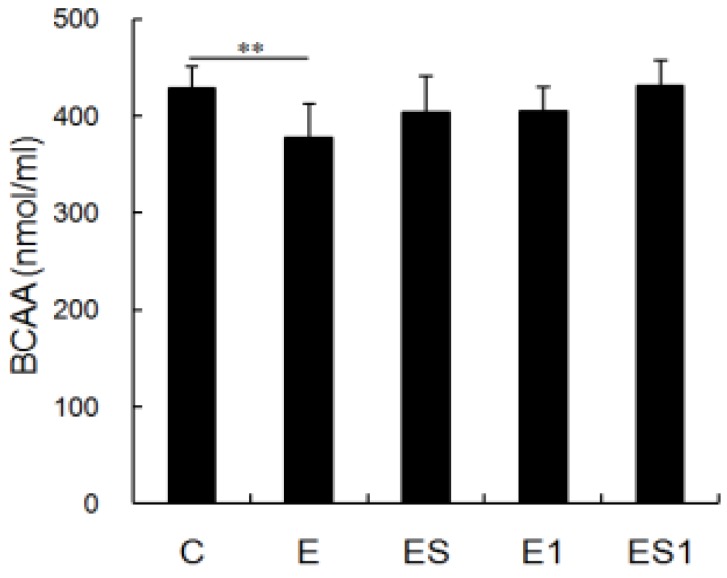
Effects of strenuous exercise and branched-chain amino acid (BCAA) supplementation on the serum BCAA concentration. BCAA levels in the serum were measured using high-performance liquid chromatography (HPLC). Data are means ± Standard Deviation (SD). ** *p* < 0.01. (C) Control; (E) Exercise; (E1) Exercise with 1 week to recover; (ES) Exercise + Supplementation; ES1) Exercise + Supplementation with 1 week to recover; the following are same.

Figure 2 shows changes in serum BCAA levels after strenuous exercise and BCAA supplementation. The data showed that serum BCAA levels of rats from strenuous exercise group decreased significantly as compared with the rats from the sedentary group (decreased by 11.71%, *p* = 0.007). On the other hand, BCAA supplementation could recover serum BCAA levels. There was no significant difference between group ES and C. After seven days of recovery, no difference was observed between group E1 and C.

### 3.3. Phagocytosis

**Figure 3:**
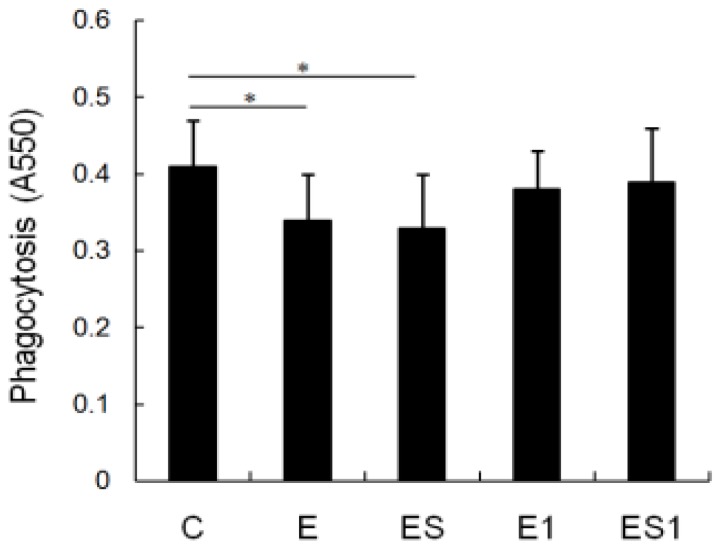
Effects of strenuous exercise and BCAA supplementation on the phagocytosis of MФs. MФs were incubated with 0.1% neutral red solution for 30 min, as described in “Materials and Methods”. The absorbance at a wavelength of 550 nm represented phagocytosis by macrophages. Data are means ± SD of A550 nm. * *p* < 0.05.

Figure 3 shows changes in phagocytosis of MФs after strenuous exercise and BCAA supplementation. A decreased capacity for uptake of the neutral red (A550 nm) was observed in MФs from the strenuous exercise group as compared with the cells from the sedentary group (Control *vs.* strenuous exercise, 0.41 ± 0.06 *vs.* 0.34 ± 0.06; decreased by 17.07%, *p* = 0.031). Furthermore, BCAA supplementation could not improve the phagocytosis of MФs (Control *vs.* ES, 0.41 ± 0.06 *vs.* 0.33 ± 0.07; decreased by 19.51%, *p* = 0.018). Meanwhile, there was no significant difference in the phagocytosis of MФs between group ES1, E1 and C.

### 3.4. Chemotaxis

**Figure 4:**
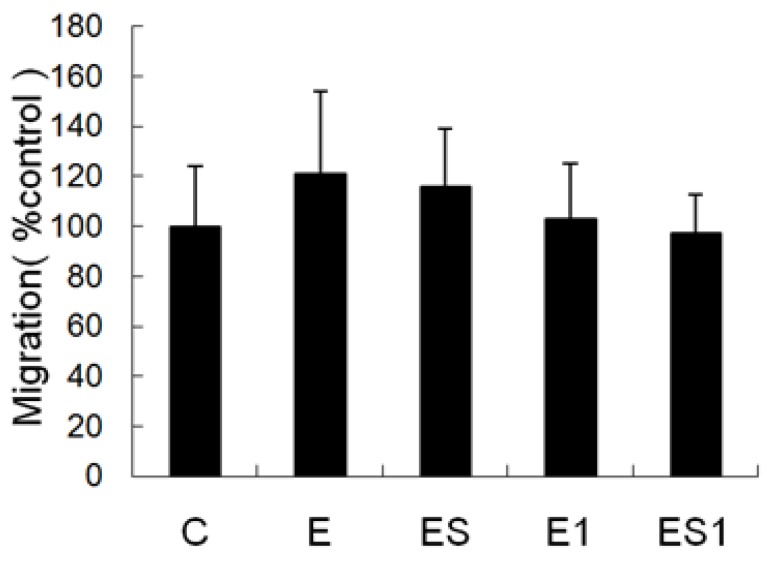
Effects of strenuous exercise and BCAA supplementation on the chemotaxis of MФs. MФs were added into the upper chambers of a 24-well transwell plate, while MCP-1 was added into the lower chambers of the transwells to induce migration. The number of migrated cells was counted. Data were expressed relative to control group cell migration. Data are means ± SD.

Figure 4 shows changes in chemotaxis of MФs after strenuous exercise and BCAA supplementation. In this study, we added MCP-1 into the lower chambers of the transwells to induce migration. Data showed that the migration capacity of MФs from the strenuous exercise group has a tendency to increase as compared with the cells from the sedentary control. However, there was no significant difference between the two groups. Furthermore, BCAA supplementation could not change the chemotaxis of MФs.

### 3.5. ROS Generation

**Figure 5:**
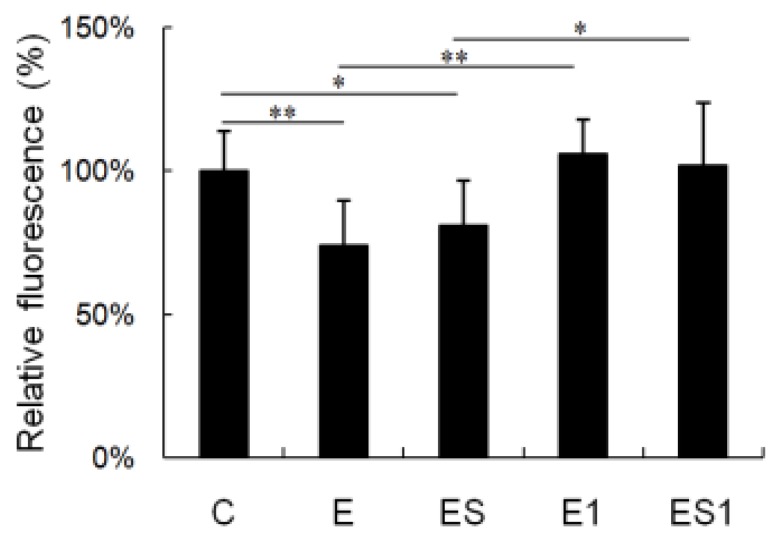
Effects of strenuous exercise and BCAA supplementation on the reactive oxygen species (ROS) generation of MФs. MФs were incubated with 2′, 7′-dichlorofluorescein diacetate (DCFH-DA). DCFH-DA oxidation was monitored by flow cytometry. Data are means ± SD of relative fluorescence intensity. * *p* < 0.05; ** *p* < 0.01.

Figure 5 shows changes in ROS generation of MФs after strenuous exercise and BCAA supplementation. The production of ROS in MФs from the strenuous exercise group decreased significantly as compared with the cells from the sedentary group (Control *vs.* Strenuous exercise, 1.00 ± 0.14 *vs.* 0.74 ± 0.16; decreased by 26%, *p* = 0.003). After seven days of recovery, the ROS generation of MФs from group E1 was significantly higher than that of group E (*p* = 0.000), and no difference was observed between group E1 and C. In addition, the ROS generation of MФs from group ES did not change as compared with group E. It was still significantly lower than that of group C (*p* = 0.020). Similarly, the ROS generation of MФs from group ES1 was significantly higher than that of group ES (*p* = 0.012), and there was no difference as compared with group E1 or C (*p* > 0.05).

### 3.6. MHC II mRNA Level

**Figure 6:**
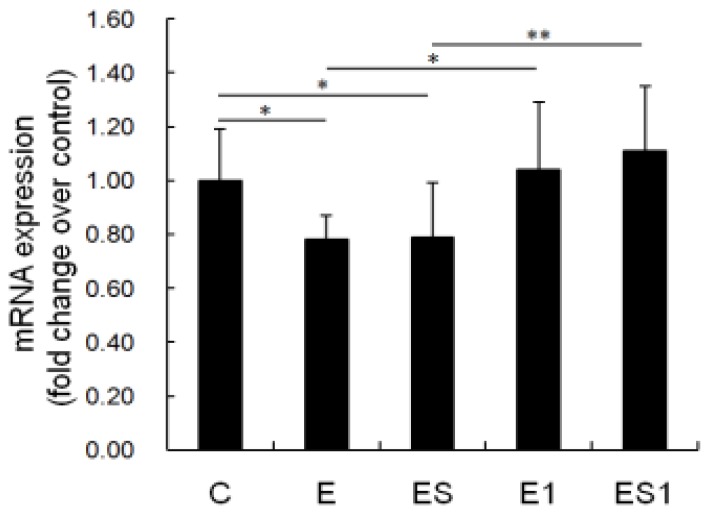
Effects of strenuous exercise and BCAA supplementation on MHC II mRNA level of MФs. Total RNA of macrophages was isolated and reverse transcribed into cDNA. Quantitative PCR was carried out using β-actin as the housekeeping gene. Data are means ± SD. * *p* < 0.05; ** *p* < 0.01.

Figure 6 shows changes in MHC II mRNA level of MФs after strenuous exercise and BCAA supplementation. Data showed that MHC II, the key molecule mediated macrophage antigen presentation [28,29,30,31], decreased significantly in the MФs of strenuous exercise rats (Control *vs.* Strenuous exercise, 1.00 ± 0.19 *vs.* 0.78 ± 0.09; decreased by 22%, *p* = 0.041). After seven days of recovery, MHC II mRNA of MФs from group E1 was significantly higher than group E (*p* = 0.017), and no difference was observed between group E1 and C. In addition, MHC II mRNA of MФs from group ES did not change as compared with that of group E. It was still significantly lower than that of group C (*p* = 0.047). Similarly, MHC II mRNA of MФs from group ES1 was significantly higher than that of group ES (*p* = 0.003), and there was no difference as compared with group E1 or C (*p* > 0.05).

## 4. Discussion

In order to investigate the effects of strenuous exercise and BCAA supplementation on blood index of rats, blood hemoglobin and serum testosterone were tested. The data showed that blood hemoglobin and serum testosterone in the strenuous exercise group decreased significantly as compared with the control group. Furthermore, the body weight of the strenuous exercise group reduced significantly than that of the sedentary control. In addition, most of experimental groups could not keep up with the velocity of treadmill and had to be assisted by hand to complete the job at the last week of training. This means that the high-intensity exercise induced them to approach exhaustion. The data showed that the unbalanced condition was induced by four weeks of high-intensity training. Moreover, BCAA supplementation could not change the body weight, the blood hemoglobin and the serum testosterone as compared with group E, which means that the unbalanced condition induced by strenuous exercise could not be improved by BCAA supplementation.

Phagocytosis, chemotaxis and antigen presentation are very important for macrophages in the removal of potentially pathogenic microorganisms [32]. Consequently, we tested the effects of strenuous exercise on the functions of MФs. The results indicated that phagocytosis capacity (decreased by 17.07%, *p* < 0.05) and major histocompatibility complex (MHC) II antigen (decreased by 22%, *p* < 0.05) of MФs from the strenuous exercise group was significantly lower than that of the control group. MHC II is a key molecule mediated macrophage antigen presentation [28,29,30,31]; meaning that MHC II mediated antigen presentation of MФs will be inhibited by strenuous exercise. Our results are similar to others. For instance, it has been reported that the phagocytosis of pulmonary alveolar macrophages (PAM) was impaired after seven weeks of strenuous exercise [33]. In the study of Woods *et al.*, exhaustive exercise can negatively affect macrophages expression of MHC II, which may be detrimental to the ability of MФs to present antigen to T lymphocytes [34]. In addition, in a previous study we found that 11 weeks of overload training decreased the phagocytosis and chemotaxis of MФs [4]. However, in this study we did not find the decrease of chemotaxis of MФs after four weeks of strenuous exercise. The varying results may be influenced by the quality and/or quantity of exercise applied in the studies.

In addition, we measured the production of intracellular reactive oxygen species (ROS) in macrophages. To investigate the effect of strenuous exercise on the production of ROS, no stimulus (e.g., Lipopolysaccharides) was added to the cell suspension to induce macrophage activation. The data showed that the production of ROS in macrophages from strenuous exercise group was significantly lower than that of the control group (decreased by 26%, *p* < 0.01). The result is similar to our previous study, in which we found that ROS production of macrophages was inhibited by 11 weeks of overload training [4]. ROS are generally considered cytotoxic. However, intracellular ROS also serves as important second messengers in cell signaling [35]. A number of studies have shown that ROS play important roles in regulating macrophages’ survival [36], differentiation [37] and secretion of inflammatory cytokines [38]. Therefore, the ROS level of macrophages from strenuous exercise group was lower than physiological levels, which would impair the function of macrophages mediated by ROS.

These results indicate that the functions (*i.e.*, phagocytosis capacity, MHC II-mediated antigen presentation and ROS generation) of macrophages were inhibited by four weeks of high-intensity exercise, which could impair the removal capability of potentially pathogenic microorganisms. This may be a mechanism that explains why long-term intensive exercise induces immunodepression and increases the susceptibility to infections. Although the functions of macrophages were impaired after strenuous exercise, these functions were nearly recovered after one week recovery. It means that the hindering functions of macrophages induced by strenuous exercise, was non-permanent and reversible.

Taking account of the good effect of BCAA supplementation on immune system [11,14,15], we speculated that BCAA supplementation would be beneficial to the macrophages of rats from strenuous exercise. The data showed that the phagocytosis, the ROS production and the MHC II mRNA of MФs from group ES was still significantly lower than that of group C. Moreover, there was no significant difference between that of groups ES and E. It means that the hindering function of MФs induced by strenuous exercise could not be ameliorated by BCAA supplementation. The results may be explained by the serum BCAA levels. In this study, we found that serum BCAA level decreased significantly in the rats of strenuous exercise. BCAA supplementation could slightly increase the serum BCAA level of rats from strenuous exercise; however, there was no significant difference between groups ES and E (increased by 6.70%, *p* > 0.05). Few studies have evaluated the roles of BCAA on macrophage function. For instance, Petro and Bhattacharjee reported that the ability of peritoneal macrophages to phagocytose and to kill *S. typhimurium* was not affected by BCAA restriction [39]. Kitagawa *et al.* also reported that BCAAs have protective effects on hepatic ischemia-reperfusion-induced liver injury through the attenuation of Kupffer cell (macrophage) activation [40]. *In vitro* experiments showed that high-BCAA medium increased IL-10 expression and phagocytic activity of microglial cells (macrophages) but did not affect the migration ability of these cells [41]. In spite of all these, our results are still difficult to compare with that of other studies owing to the fact that little attention has been paid to the effects of BCAA supplementation on peritoneal macrophage functions, especially in the model of strenuous exercise. If the data from rats are similar to human beings, then our results suggest that dietary BCAA supplementation is not useful to improving the macrophages function of people who engage in strenuous exercise. However, the effects of BCAA supplementation may be affected by many factors such as the dosing and timing. It is currently unknown whether the observed results could be affected by BCAA supplementation in the pre-workout period or at different doses.

## 5. Conclusions

Strenuous exercise impaired phagocytosis capacity, MHC II-mediated antigen presentation and ROS generation of macrophages. This may be a mechanism that explains why strenuous exercise induces immunodepression and increases the susceptibility to infections. Moreover, the impaired function of macrophage induced by strenuous exercise could not be ameliorated by BCAA supplementation in the dosing and timing used for this study.
